# The New Call to the Medical Profession

**Published:** 1917-04-28

**Authors:** 


### THE NEW CALL TO THE MEDICAL PROFESSION.

The war has brought to the medical profession in relation to the national cause a special position of honour and of opportunity, and from this position there necessarily arises a corresponding measure of obligation and of duty. The demands made upon the profession have steadily increased, and recently it has become manifest that satisfaction of the full wishes of the Army must mean prejudice to the medical needs of the civil population. It is certain that the Army has not received as many medical officers as the authorities desire, but it is not less certain that members of the general public have suffered from the absence of so many of their usual medical advisers. In short, it has become evident that there are not enough doctors "to go round," and that neither the demands of the Army nor the needs of the civil population can be met to the full extent which an ideal scheme would contemplate. The "cloth" is limited, and the "coat" must therefore be cut on a scale less ambitious than the best judgment would dictate. The various Medical War Committees have been busy with the task of accommodation for some months, and a most ungrateful task it has proved to be. To give satisfaction to everybody concerned in such an undertaking was obviously impossible from the outset, and probably by this time most members of these committees have learned that kicks rather than halfpence are to be their main reward. We express no judgment on any individual decision which has been reached when we say that those who have undertaken this necessary and delicate work have made a substantial contribution to the national cause, and have deserved well alike of their own profession and of the State. Their critics and censors are on every hand, and we will not say that all their proceedings have commanded our sympathy. But we recognise both the difficulty of their task and the loyalty of their service, and we are sure that they merit much more gratitude and goodwill than they have received.

The anxieties of the position are greatly increased by the conditions described in the letter addressed to the profession by the Secretary for War at the desire of the War Cabinet. The enemy, we are told, "have deliberately instituted a submarine campaign against hospital ships," and it has therefore "become essential that a large number of hospitals should be established overseas in the various theatres of war for the treatment of the sick and wounded." Such a situation obviously imposes a duty upon the Government, and this duty they propose to meet in the manner Lord Derby describes. Their interpretation must necessarily command respect, though, truth to tell, it seems somewhat strange that in view of the efficient protection that is being afforded to transport and other vessels engaged in cross-Channel traffic, similar protection cannot be afforded to hospital ships. Such a question is bound to be asked, though there may be reasons why it cannot safely be answered. No one would propose that any risk which can be avoided should be allowed to apply to wounded and helpless men, and if the authorities decide that the only way to avoid dangers of this order is to establish more hospitals in the various theatres of war this decision must be accepted. The time is not one for minute criticism or for pertinacious questionings. Rather we must listen to the voice of authority, and trust it is instructed by sound judgment. At the same time it must be remembered that the claim advanced on the medical profession is a very severe one, and that it is imposed on shoulders which are already carrying a heavy burden. The profession, we are certain, is willing and ready to do whatever is possible, but in some directions at all events the limit of the possible has almost been reached. There is in the terms of Lord Derby's letter a generous recognition of what has already been sacrificed both by doctors and by civilian patients, and a frank acknowledgment of the severity of the new demands. But not less decided is the statement of the hard fact that if wounded men are to receive adequate treatment without exposure to the risk of the diabolical submarine campaign more hospitals must be established abroad, and more doctors must be obtained to man them. This is the new development which the medical profession is now called upon to face.

On medical men of military age the authorities have a ready hold. These, like other citizens, come under the Military Service Acts. That is, they can be called to take service in the Army by force of law. It therefore follows that the War Office by a stroke of the pen can at once recruit all medical men who are under forty-one years of age, and who yet remain in civil practice. Were this power exercised there are certain districts of the country which would be practically deprived of medical aid. On grounds of humanity the War Office naturally desires to avoid this result, but it must be remembered that the first duty of the military authorities is to the soldiers, and that they have a legal right to enforce their claim. They hold that with efficient organisation of the senior men substitutes can be found for the men of military age, and Lord Derby's letter is a call for the prompt establishment of this organisation. Hence every medical man over military age is invited to register his name with the local Medical War Committee, and to engage to undertake any work which is within his capacity and which will help to set free a junior *confrère*. It seems to us that this invitation must in the circumstances be accepted. The country needs our help, and such an appeal cannot fail of a generous response.

